# Cancer-Associated Fibroblasts Confer Gemcitabine Resistance to Pancreatic Cancer Cells through PTEN-Targeting miRNAs in Exosomes

**DOI:** 10.3390/cancers14112812

**Published:** 2022-06-06

**Authors:** Katherine E. Richards, Weikun Xiao, Reginald Hill, on behalf of the USC Pancreas Research Team

**Affiliations:** 1 Department of Biological Sciences, University of Notre Dame, Notre Dame, IN 45556, USA; krichar8@nd.edu; 2 Harper Cancer Research Institute, University of Notre Dame, Notre Dame, IN 46617, USA; 3 Lawrence J. Ellison Institute of Transformative Medicine, Los Angeles, CA 90064, USA; wxiao@eitm.org; 4 Keck School of Medicine of USC, University of Southern California, Los Angeles, CA 90033, USA

**Keywords:** fibroblasts, exosomes, miRNAs, pancreatic cancer, PTEN

## Abstract

**Simple Summary:**

Previous studies have shown that cancer associated fibroblasts exposed to chemotherapy release exosomes which promote chemoresistance in recipient cells. However, the molecular mechanism responsible for this has not been fully elucidated. In this study, we found that gemcitabine treatment caused fibroblasts to release exosome which contain PTEN-targeting miRNAs. These findings shed light on how fibroblasts exposed to chemotherapy promote tumor growth and drug resistance.

**Abstract:**

Pancreatic ductal adenocarcinoma (PDAC) is currently the third leading cause of cancer-related death in the United States. Even though the poor prognosis of PDAC is often attributed to late diagnosis, patients with an early diagnosis who undergo tumor resection and adjuvant chemotherapy still show tumor recurrence, highlighting a need to develop therapies which can overcome chemoresistance. Chemoresistance has been linked to the high expression of microRNAs (miRs), such as miR-21, within tumor cells. Tumor cells can collect miRs through the uptake of miR-containing lipid extracellular vesicles called exosomes. These exosomes are secreted in high numbers from cancer-associated fibroblasts (CAFs) within the tumor microenvironment during gemcitabine treatment and can contribute to cell proliferation and chemoresistance. Here, we show a novel mechanism in which CAF-derived exosomes may promote proliferation and chemoresistance, in part, through suppression of the tumor suppressor PTEN. We identified five microRNAs: miR-21, miR-181a, miR-221, miR-222, and miR-92a, that significantly increased in number within the CAF exosomes secreted during gemcitabine treatment which target PTEN. Furthermore, we found that CAF exosomes suppressed PTEN expression in vitro and that treatment with the exosome inhibitor GW4869 blocked PTEN suppression in vivo. Collectively, these findings highlight a mechanism through which the PTEN expression loss, often seen in PDAC, may be attained and lend support to investigations into the use of exosome inhibitors as potential therapeutics to improve the effectiveness of chemotherapy.

## 1. Introduction

Pancreatic ductal adenocarcinoma (PDAC) is currently the third leading cause of cancer-related deaths in the United States with a 5-year survival rate of 11% [1] and is projected to become the second leading cause of cancer-related deaths by 2030 [2]. Despite the promise of targeted therapies [3], gemcitabine-based chemotherapy or FOLFIRINOX is still the mainstream first-line chemotherapy for advanced pancreatic cancer [4]. Gemcitabine is often administered both prior to and post tumor resection. However, 74% of PDAC patients with resected tumors given adjuvant gemcitabine eventually show tumor recurrence [5]. This demonstrates the ability of PDAC cells to overcome gemcitabine-based chemotherapy and highlights the need to understand the biology underlying resistance to gemcitabine in PDAC.

In order to better treat patients, we need a more thorough understanding of the entire tumor mass. The majority of cells in the tumor are stromal cells which contribute to the hallmark desmoplasia of the disease [6,7]. Of the many cell types that make up the stroma, cancer-associated fibroblasts (CAFs) are one of the most prevalent and significant populations of cells due to the proven role they play in the promotion of tumor growth and chemoresistance [8,9,10]. Mechanisms of tumor progression previously attributed to CAFs include the secretion of cytokines [8] and growth factors [11,12]. However, recent efforts show that microRNA-containing extracellular vesicles, such as exosomes, which can travel through the bloodstream to distant organs [13], are secreted by CAFs and promote PDAC cell proliferation and chemoresistance [14,15,16] as well as fostering metastasis [17]. Furthermore, CAFs hypersecrete tumor-promoting exosomes in response to gemcitabine treatment and inhibit this hypersecretion in vivo attenuated tumor growth, pointing to a mechanism through which CAF derived exosomes may prime PDAC cells to become resistant to chemotherapy [18]. Despite these initial findings, the molecular mechanisms underlying the ability of gemcitabine treated CAFs to promote tumor growth and chemoresistance in PDAC cells requires further elucidation.

Given the critical need to gain more insight into how this exosome-based chemoresistance may be hindering current treatments, we set out to uncover the miRNA pathways that are differently expressed in CAFs that were exposed to gemcitabine. We hypothesized that identifying miRNAs that play a critical role in this tumor microenvironment PDAC cell crosstalk may give us important information that can aid strategy development for overcoming CAF-mediated resistance to gemcitabine. In this study, we identified five oncogenic microRNAs (oncomiRs) that are upregulated and hypersecreted through CAF-derived exosomes during gemcitabine treatment. All five microRNAs are associated with increased proliferation and inhibited apoptosis, targeting the tumor suppressor phosphatase and tensin homolog (PTEN). PTEN is usually altered in PDAC patients [19], and genetic studies have demonstrated that a loss of PTEN expression promotes PDAC progression [20,21]. Moreover, recent data show that PTEN can be dysregulated in both PDAC cancer cells and stromal cells through posttranscriptional modification and nongenomic regulation [22]. Our results showed that CAFs exposed to gemcitabine release *PTEN*-targeting miRNAs, aiding both tumor growth and chemoresistance. Suppression of exosome secretion reduced PTEN-targeting miRNA and restored PTEN expression in vivo. Together, these findings suggest that CAF-derived exosomes play an important role in the regulation of PTEN expression in PDAC and reinforce the exploration of exosome inhibitors as a potential therapeutic option for patients with this disease.

## 2. Materials and Methods

### 2.1. Cell Culture

AsPC1 cells were purchased from ATCC. Dr. Timothy Donahue (University of California, Los Angeles, CA, USA) provided the L3.6 cells. Fibroblast cell lines were donated by Dr. Melissa Fishel (IU Simon Cancer Research Center), and were immortalized and tested as previously described [18]. In this study, CAF1 cells refer to UH1301-63 cells, and CAF2 cells refer to UH1303-02 cells. L3.6 cells and fibroblasts were grown in DMEM (Sigma, St. Louis, MO, USA), and AsPC1 cells were grown in RPMI (Sigma, St. Louis, MO, USA), according to standard procedures and protocols supplemented with 10% FBS (RMBIO, Missoula, Montana) and 1% Pen-Strep (Life Technologies, Carlsbad, CA, USA). Presence of mycoplasma within conditioned-cell media was tested throughout the studies using the MycoAlert™ kit (Lonza, Basel, Switzerland). AsPC1 cell authentication was performed by Genetica DNA Laboratories, showing a 97–100% match to the correct cell line in both ATCC and DSMZ databases. Because the L3.6 and fibroblasts were not commercial cell lines, they were not part of any databases, yet they did not match the genetic profiles of any cell line within said databases, suggesting no cell-cell contamination had occurred. To determine that the primary fibroblast cell lines were not derived from cancer cells, Sanger sequencing was performed for the KRAS exon 2 locus as previously described [18].

#### 2.1.1. Conditioned-Cell Media Transfer

CAFs were plated at 1 million cells/flask in exosome free media, and conditioned media was collected and spun down at 1200 RPM for 5 min with the supernatant spun down at 16,500× *g* for 20 min. For the generation of exosome-depleted conditioned media, conditioned media was spun down at 1200 RPM for 5 min, and the supernatant was spun down at 16,500× *g* for 20 min, as well as 120,000× *g* for 70 min prior to filtration to deplete the media of exosomes without the depletion of proteins. Cells were grown in conditioned media, conditioned and exosome-depleted media, and control media for four days, treated with new conditioned media each day.

#### 2.1.2. Co-Culture Studies

CAFs were plated on 12-well Transwell^®^ polyester permeable supports (Corning Inc., Cornyn, NY, USA) with 0.4 µm pore size. CAFs on the permeable supports were treated with GW4869 (20 µM) or DMSO and cocultured over AsPC1 cells in a 12-well plate for 3 days. GW4869 was dissolved in DMSO to create a working stock solution of 5 mM GW4869. This working solution was used to achieve a final 20 μM GW4869 concentration in cell culture media.

### 2.2. Exosome Isolation, RNA Extraction, and MicroRNA-Seq

Fibroblasts were grown in exosome-free media and 20 mL of cell-conditioned media was collected, and exosomes were isolated using ExoQuick-TC™ reagent. Exosome RNA was extracted via standard Trizol^®^ method. microRNA-Seq was performed by the Notre Dame Genomics and Bioinformatics Core.

### 2.3. Target Gene Prediction and Pathway Analysis

Experimentally validated gene targets from TarBase within cellular pathways targeted by all five microRNAs were identified via DIANA TOOLS miRPath software (University of Thessaly, Volos, Greece). Predicted microRNA target gene scores were derived from DIANA TOOLS microT-CDS target prediction analysis software.

### 2.4. RNA Isolation and RT-PCR

#### 2.4.1. Exosome MicroRNA

Fibroblasts were plated at 1 million cells/flask with 20 mL of exosome-free media and treated for 3 days with 1 µM gemcitabine or PBS. An amount of 10 mL of cell-conditioned media was collected, and exosomes were isolated as described herein. RNA from exosome pellets was extracted with Trizol^®^ as described herein, and reverse transcription and RT-PCR was performed using miScript II RT Kit and miScript SYBR^®^ Green PCR kit (QIAGEN, Hilden, Germany) according to the manufacturer’s protocol. Samples were normalized by volume of conditioned-cell media. microRNA primer assays were purchased from QIAGEN.

#### 2.4.2. Cellular MicroRNA and mRNA

RNA was extracted from cells using the Trizol^®^ standard protocol. miScript II RT Kit and miScript SYBR^®^ Green PCR kit (QIAGEN, Hilden, Germany), as well as primer assays from QIAGEN, were used for quantification of cellular microRNA levels. QIAGEN SYBR^®^ Green QuantiFast RT-PCR kit and primer assays from QIAGEN were utilized for the quantification of cellular mRNA levels. Manufacturer’s protocols were used. microRNA was normalized to RNU6 and mRNA was normalized to GAPDH. Amplification and quantification were performed with Bio-Rad CFX Connect™ Real-Time PCR Detection System.

### 2.5. Mimic Transfection

MicroRNA mimics were purchased from QIAGEN. Briefly, cells were plated with 10nM microRNA mimic or negative control siRNA (QIAGEN, Hilden, Germany) along with 0.6% HiPerFect reagent. Cells were collected for RT-PCR 48 h post transfection. Manufacture’s protocol and guidelines were used.

### 2.6. Mouse Studies

All animal studies were done under the IACUC approved protocol number 16-03-3033 at the University of Notre Dame. NOD/SCID mice were subcutaneously implanted with 1 million AsPC1 cells and 200,000 CAF1 cells. After two weeks, post implantation, mice were treated intraperitoneally with DMSO + PBS, DMSO + gemcitabine (GEM 50 mg/kg) or GEM + GW4869 (2.5 µg/g), twice weekly for two weeks. Posttreatment mice were euthanized, and tumors were excised and partly flash frozen. For RT-PCR, tumor tissue was ground and resuspended in Trizol^®^ for RNA isolation according to the manufacturer’s protocol. RT-PCR was performed with QIAGEN SYBR^®^ Green QuantiFast RT-PCR kit and primer assays from QIAGEN. Data was normalized to GAPDH.

### 2.7. Western Blot

Cells were lysed in Laemmli buffer, subjected to gel electrophoresis (Bio-Rad Mini Protean TGX Gel 400091313, Bio-Rad Laboratories, Hercules, CA, USA) and membrane transfer. Membranes were blocked in 5% dry milk/TBS-T, incubated with primary antibodies (PTEN:138G6; p-AKT: S473 (Cell Signaling Technology, Inc., Danvers, MA, USA) diluted in 1% dry milk/TBS-T overnight, β-actin 3177S (Cell Signaling Technology, Inc., Danvers, MA, USA); incubated with secondary antibody 7074S (Cell Signaling Technology, Inc., Danvers, MA, USA), diluted in 1% dry milk/TBS-T for 1–2 h, washed with TBS-T, and subjected to an enhanced chemiluminescent substrate (SuperSignal West Dura; Thermo Fisher, Waltham, MA, USA). Protein levels were detected by enhanced chemiluminescence (ECL; Thermo Scientific 32106). Protein levels were normalized and quantified using ImageJ software.

### 2.8. Statistical Analysis

All comparisons will be analyzed as mean ± standard deviation (SD), *n* = 3. Statistical analyses will be performed by unpaired Students *t*-test. Significance will be defined as * *p* < 0.05 and ** *p* < 0.01.

## 3. Results

### 3.1. Gemcitabine Exposure Increases Expression of Five OncomiRs within Pancreatic CAF Exosomes

Due to the fact that CAFs rapidly produce exosomes in response to gemcitabine treatment [16,18] and that CAF exosomes promote the proliferation and chemoresistance of PDAC cells [14,18], we hypothesized that exosome encased factors important for propagating chemoresistance may increase in number during gemcitabine treatment. Therefore, we treated PDAC CAFs with gemcitabine or PBS and isolated the CAF-secreted exosomes (from CAF-conditioned media) to identify the factors within CAF exosomes which may be responsible for eliciting proliferation and chemoresistance, further elucidating the biological mechanism behind these exosome-induced phenotypes.

**Figure 1:**
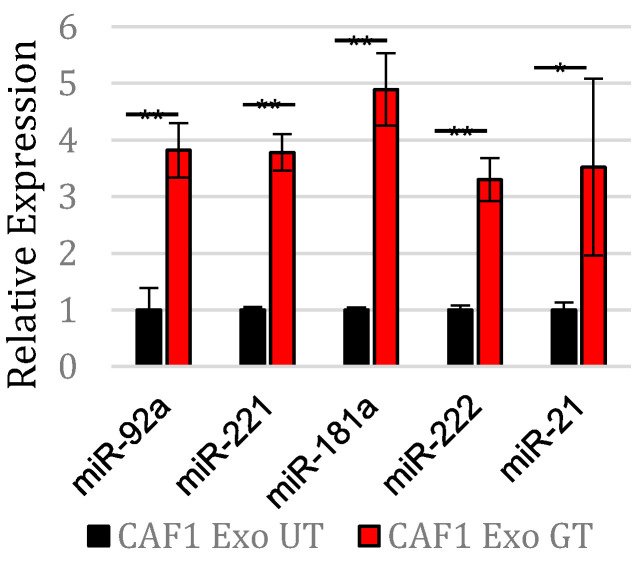
Expression of identified exosomal miRs in CAFs exposed to gemcitabine.RT-PCR normalized expression of miRs from exosomes in CAFs ex-posed to gemcitabine (Exo GT) and untreated CAFs (Exo UT). ** *p* < 0.01. * *p* < 0.05.

We first validated the presence of exosomes using transmission electron microscopy, western blotting, and dynamic light scattering (Figure S1) as previously described [18]. Instead of posttranslationally altering the protein function [23], we decided to perform a microRNA-Seq, which normalizes the quantification data to total RNA, to quantify the relative copy numbers of microRNAs inside the exosome populations secreted from the control CAFs vs. the gemcitabine-treated CAFs. This decision was also based on the fact that exosomes are rich in microRNAs [24] that have a robust ability to alter cell phenotype through blocking protein translation. The results of the microRNA-Seq showed several microRNAs being highly secreted during the gemcitabine treatment, including miR-92a, miR-21, miR-181a, miR-221, and miR-222 (Table 1). We validated the increase in copy numbers of these microRNAs within the gemcitabine-treated CAF-derived exosomes via RT-PCR (Figure 1).

### 3.2. PTEN Is the Predicted Target of miRNAs Released by Gemcitabine-Treated CAF-Derived Exosomes

It has been shown that miRNAs can control a wide range of cellular functions and target hundreds of genes [25]. To overcome this issue, we utilized DIANA TOOLS miRPath software to identify any potential targets of the five identified microRNAs using both bioinformatics sequence pairing and experimental data. Several cellular pathways important to tumor biology are commonly targeted by all five microRNAs, including the Wnt, MAPK, and PI3K/AKT signaling pathways (Table S1). One such targeted gene from the PI3K/AKT pathway is the tumor suppressor gene PTEN. We decided to focus on PTEN because our past studies had shown that PTEN loss accelerates PDAC development [21], and literature has established the alterations of the PTEN/PI3K/AKT pathway are a mechanism for mediating drug resistance [26]. Using DIANA TOOLS microT-CDS target prediction analysis software, we found that PTEN was predicted to be a target of miR-92a, miR-21, miR-181a, miR-221, and miR-222 (Table 2). Moreover, all have been linked with the ability to suppress PTEN functions [27,28,29,30]. Taken together, this led us to focus on the PTEN expression in our experimental system.

### 3.3. PTEN Levels Are Suppressed by Exosomes from CAFs

**Figure 2:**
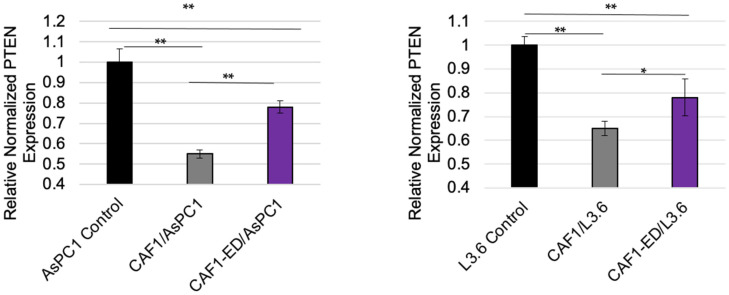
PTEN expression in cells where exosomes are retained or depleted from CAF-derived media. PTEN expression was analyzed via RT-PCR in AsPC1 cells (left) or L3.6 cells (right) after cultured in normal DMEM or RPMI (control), CAF-conditioned media, or exosome-depleted CAF-conditioned media (CAF1-ED) each day for four days. ** *p* < 0.01. * *p* < 0.05.

To ascertain whether CAF exosomes can affect PTEN levels in vitro, PDAC cells were cultured in control media, CAF-conditioned media, and exosome-depleted CAF-conditioned media, with the PTEN levels then quantified. The CAF-conditioned media showed a decrease in the PTEN mRNA and protein quantities in comparison with the control media. However, the PDAC cells grown in the CAF-conditioned media, with some of the exosomes removed via ultracentrifugation, displayed significantly higher PTEN quantities compared to the CAF-conditioned media with no exosome removal via RT-PCR (Figure 2). This was supported by western blot data showing the same trend (Supplemental Figure S2), suggesting that factors within GEM-treated CAF-derived exosomes may be, in part, altering PTEN. Because PTEN inhibits the phosphorylation and activation of AKT, we analyzed the pAKT levels in these cells by western blot. CAF-conditioned media increased the pAKT protein levels in PDAC cells, but the pAKT levels did not increase in the exosome-depleted CAF-conditioned media, indicating that there was a loss of PTEN functionality in the PDAC cells exposed to CAF-derived exosomes (Figure S2).

### 3.4. Exosome Inhibition Restores PTEN Expression to Tumors Treated with Gemcitabine In Vivo

**Figure 3:**
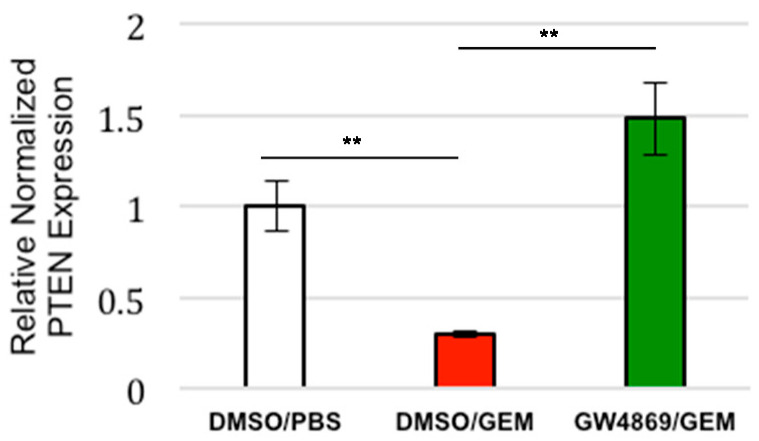
Evaluation of the effect of GEM treatment and exosome inhibition on PTEN expression in in vivo PDAC model. Mice with subcutaneous tumors (PDACs+CAFs) were treated for two weeks with PBS, Gemcitabine, or GW4869/Gemcitabine, and their PTEN mRNA levels were quantified via RT-PCR. ** *p* < 0.01.

Our previous study utilized mice that were coinjected subcutaneously with AsPC1 cells and CAFs to test the effect that the exosome inhibitor GW4869 had on tumor growth. Tumors in the control mice, and the mice treated with GEM alone, steadily increased in size over time, while the tumors in mice that were given combination therapy (GW4869 and GEM) remained relatively similar in size, displaying significantly reduced growth compared to the control mice [18]. Based on those findings, we performed a follow-up experiment to assess if the inhibiting exosome secretion in vivo had affected the PTEN expression in those tumors. Thus, we examined the samples from the aforementioned study. The RT-PCR analysis showed that the tumors from the mice that were given gemcitabine alone displayed significantly reduced PTEN expression; however, PTEN expression was restored in the tumors of the mice given combined treatment of gemcitabine and GW4869 (Figure 3). Together these results show that CAF-derived exosome signaling plays a role in the suppression of PTEN expression often seen in PDAC.

### 3.5. MicroRNA-92a Targets PTEN mRNA in PDAC Cells and Is Distributed through CAF Exosomes

**Figure 4:**
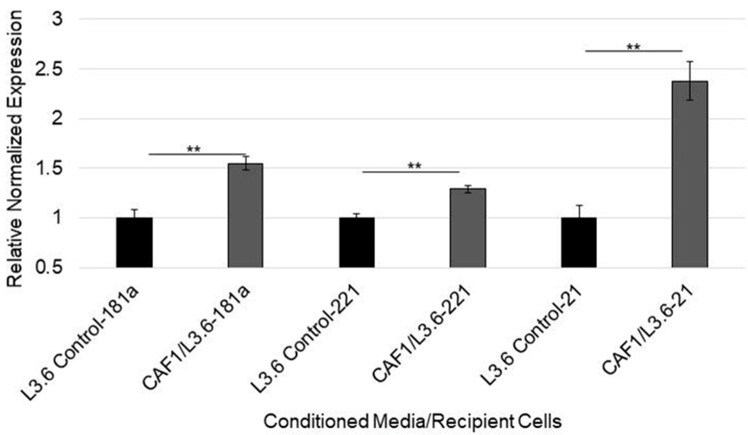
Expression of PTEN-targeting miRs in cells exposed to CAF-conditioned media. L3.6 epithelial cells were cultured in DMEM or gemcitabine-treated CAF-conditioned media for four days. Expression of microRNAs within recipient L3.6 cells was thereafter quantified via RT-PCR. ** *p* < 0.01.

**Figure 5:**
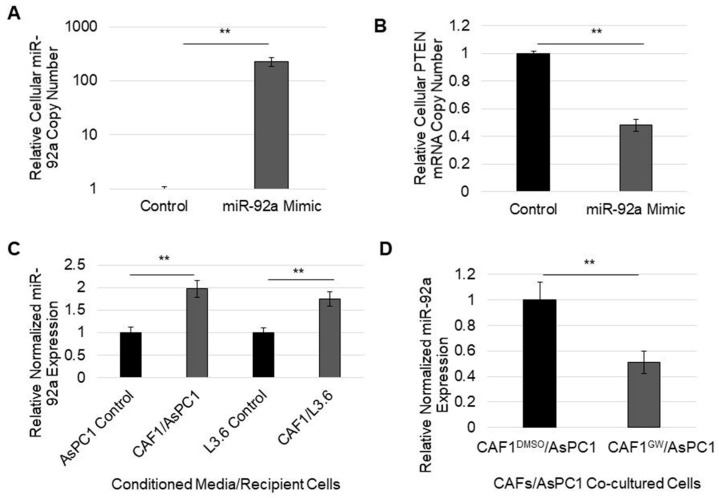
MicroRNA-92a expression in cells exposed to CAF-derived exosomes. (**A**) validation of mir-92a expression in a mimic-containing cell line (AsPC1). (**B**) PTEN mRNA levels were measured in AsPC1 cells transfected with miR-92a mimic compared to AsPC1 cells transfected with scramble negative control miR via RT-PCR. (**C**) miR-92a expression levels were quantified via RT-PCR within cells which received control or GEM-treated, CAF-conditioned media for four days. (**D**) AsPC1 cells were co-cultured underneath GEM-treated CAFs or GEM+GW4869-treated CAFs for three days. miR-92a expression within AsPC1 cells was thereafter quantified via RT-PCR. ** *p* < 0.01.

Because PDAC cells that are exposed to CAF exosomes exhibit a decrease in PTEN expression, we sought to verify if PTEN was targeted through exosome microRNAs. We first assessed whether those same cells, with attenuated PTEN expression, had displayed increased levels of PTEN-targeting microRNAs. Indeed, when chemosensitive L3.6 cells were cultured in CAF-conditioned media, cellular levels of miR-21, miR-221, and miR-181a increased (Figure 4). To ascertain which of the five identified microRNAs may have most effectively targeted PTEN, we utilized the microRNA target scores of each using DIANA microT-CDS target prediction algorithms. Interestingly, the most understudied of the five oncomiRs, miR-92a, had the highest predicted PTEN mRNA target score of 0.971 (Table 2). Therefore, we tested to see if the transfection of miR-92a alone would decrease PTEN mRNA levels. Indeed, transfection with miR-92a significantly decreased PTEN mRNA levels by about 50% in AsPC1 cells (Figure 5A,B). Furthermore, miR-92a levels were significantly increased in the PDAC cells cultured in CAF-conditioned media compared to those in control media (Figure 5C).

To elucidate if the PTEN targeting of microRNA-92a is transferable to PDAC cells through CAF exosomes, CAFs were grown on exosome-permeable membranes with AsPC1 cells in 12-well plates while being treated with DMSO (control) or the exosome inhibitor GW4869. When CAFs were treated with GW4869, cocultured AsPC1 cells showed a reduced expression of miR-92a, showing that exosomes derived from CAFs can affect the level of miR-92a and subsequently PTEN expression in neighboring cancer cells (Figure 5D). Together, these data indicate that the delivery of microRNAs to PDAC cells through CAF exosomes may play an important role in the suppression of PTEN, often seen in PDAC. Additionally, gemcitabine, which increases exosome production, may contribute to PTEN suppression by increasing the delivery of prolific PTEN-targeting microRNAs such as microRNA-92a.

## 4. Discussion

Gemcitabine-based therapy is still a commonly utilized treatment for PDAC patients. Despite its constant utilization, the majority of patients receiving this chemotherapy will relapse [5]. Hence, the elucidation of the molecular mechanism responsible for gemcitabine resistance is of critical importance. To fully address this challenge, we must look at resistance mechanisms that arise not only from the tumor cells themselves but also from the many supporting cells that make up the desmoplastic microenvironment which is a hallmark of PDAC [31]. In our previous study, we identified a previously unknown mechanism through which CAFs exposed to gemcitabine hypersecrete exosomes, promoting tumor proliferation and chemoresistance in the recipient PDAC cells [18]. We showed that the tumors in the control mice, and the mice treated with GEM alone, steadily increased in size over time, while the tumors in mice given GEM plus an exosome inhibitor displayed a significantly reduced growth rate compared to those in the control mice [18].

We have expanded upon those studies here to identify the miRNAs inside these CAF-derived exosomes and investigate one of the major pathways they target. We identified five miRNAs that were overexpressed in the exosomes released by CAFs in response to gemcitabine treatment. The overexpression of these five miRNAs in our CAFs was validated utilizing pathway analysis to identify their targeting of PTEN. We showed that these CAF-derived exosomes suppress PTEN expression in recipient cancer cells. Using in vitro and in vivo studies, we determined that the depletion of these CAF-derived exosomes restored PTEN expression, which correlates to the suppression of tumor growth and restoration of chemosensitivity [18]. This data adds to the growing evidence that CAFs have a critical role in the transfer of chemoresistance-promoting and PTEN-targeting microRNAs [32].

Although PTEN downregulation is observed in pancreatic cancer [19] and is associated with poor prognosis and tumor recurrence [33], the mechanisms actively driving PTEN downregulation, especially in cells that make up the tumor microenvironment [22], require further elucidation [34]. Nevertheless, PTEN expression in metastatic breast cancer cells was demonstrated to be reduced through the glial exosome-delivery of microRNAs, including miR-92a [35]. Similarly, hypoxic pancreatic stellate cell-derived exosomal miRs promote the proliferation and invasion of pancreatic cancer through the PTEN/AKT pathway [36]. Despite not being CAF-derived, tumor cell-derived miR-93 was shown to also promote gemcitabine resistance through PTEN/AKT signaling [37]. Although microRNA-92a has been described as an oncomiR for breast, colon, and other cancers [38], the role of microRNA-92a in pancreatic cancer progression has not been fully explored.

**Figure 6:**
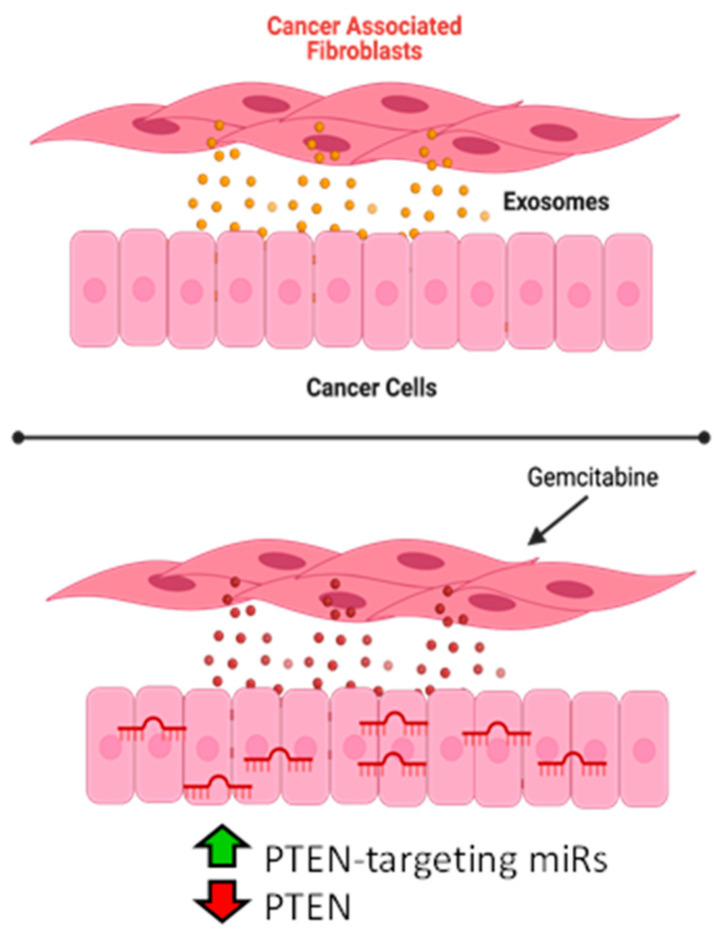
Schematic of proposed mechanism. CAFs routinely release exosomes which are taken up by cancer cells. CAFs treated with gemcitabine release exosomes which have increased expression of PTEN-targeting miRs. Cells that take up these exosomes have increased expression of these miRs and reduced expression of PTEN. Blockade of exosome release restores PTEN ex-pression.

In this study, we showed that microRNA-92a targets PTEN and is highly secreted through CAF-derived exosomes during gemcitabine treatment, overexpressed in PDAC cells cultured in CAF-conditioned media, and suppressed by the treatment of CAFs with an exosome secretion inhibitor. Thus, we showed a novel PTEN-suppression mechanism in PDAC whereby microRNAs, such as miR-92a, degrade PTEN mRNA and are delivered to PDAC cells through CAF-derived exosomes. Overall, this exosome delivery of CAF-derived oncomiRs may play a significant role in the onset of chemoresistance in patients. (Figure 6).

While we define these miRs as “PTEN-targeting”, we know that all of them target other genes that play important roles in tumorigenesis. For example, recent work has shown that miR-221 promotes the stemness of breast cancer cells by targeting DNMT3b [39], and the exosomal transfer of stroma-derived miR21 confers chemoresistance in ovarian cancer cells through targeting APAF1 [40]. Hence, in addition to PTEN, future studies should analyze the role these other known targets play in CAF-mediated growth and chemoresistance.

In addition to PTEN, exosomal miRNAs that target p53 and other genes were recently found to be increased CAFs that were exposed to gemcitabine [41] or nab paclitaxel [42]. Because of the overwhelming evidence that cancer cell exosomes promote metastasis [43,44] and drug resistance [18,40,45], focus has shifted to testing the use of exosome inhibitors to attenuate metastasis or tumor growth. The blockade of exosome signaling in vivo decreases tumor growth in pancreatic [18], prostate [46], and lung cancer [47]. Our studies utilize the neutral sphingomyelinase (nSMase) inhibitor GW4869 [48] to block exosome release. Because this compound is not approved for patient use, there are concerns about toxicity. However, we did not experience any issues with toxicity in the animals we treated [18]. Moreover, no issues were reported in a previous study utilizing a different animal model [49]. However, this could be due to the short duration of these in vivo experiments. Thus, in the future, other compounds known to block exosome release, including some already approved for other indications [50], will likely be needed for testing in longer in vivo studies. Moreover, we believe that CAF-derived miRNAs could be valuable as biomarkers for assessing how effective exosome inhibitors are at attenuating tumor growth and/or metastasis in studies, considering miRs that target PTEN, like miR-21, are known to be overexpressed in PDAC cells [51], and also that miR-21 was found to be part of a miRNA signature that could distinguish PDAC from normal control samples [52].

In addition to RNA expression changes, it is also important to note that changes to the secreted exosome proteome also play a critical role in sensitizing cells to chemotherapy, as shown by recent studies which found that increasing the efficacy of gemcitabine was possible through a mechanism that disrupted glutamine metabolic pathways [53,54]. In addition to such cell-intrinsic mechanisms, other CAF-based alterations including changes in circular RNAs (circRNAs) have been shown to play a role in the chemoresistance in PDAC cells [55]. CircRNAs acting as sponges are also known to promote gastric cancer progression via the AKT pathway [56].

Despite our focus on CAFs, the role of exosomes from immune cells cannot be overlooked. Macrophage-derived exosomes have been shown to induce different effects in cancer progression through a similar mechanism to that which we have described. Exosomal miR-21 from macrophages is linked to cisplatin resistance in gastric cancer [57]. Exosomal miRs from tumor cells can also affect macrophage polarization through the alteration of the PTEN pathway [58]. Thus, the role of exosomal signaling as a part of the complex tumor microenvironment still requires much more research.

In summary, we showed that CAFs treated with gemcitabine released exosomal miRNAs which suppress PTEN expression in the recipient PDAC cells. These findings highlight the importance of elucidating mechanisms which modulate PTEN expression in stromal cells and the role of CAF-derived exosomes in promoting chemoresistance in PDAC.

## Figures and Tables

**Table 1:** Exosomal microRNAs significantly increased in cancer associated fibroblasts treated with gemcitabine (GEM) compared to non-treated cancer associated fibroblasts (NT). ** *p* < 0.01. * *p* < 0.05.

MicroRNA	Fold Change (GEM vs. NT)	log2 Fold Change (GEM vs. NT)	*p*-Value
hsa-miR-92a-3p **	4.65992105	2.22030551	0.0042333
hsa-miR-221-3p **	4.32490131	2.11266721	0.0064948
hsa-miR-181a-5p *	3.50850376	1.81085591	0.0132411
hsa-miR-222-3p *	2.64868585	1.40527674	0.0426011
hsa-miR-21-5p **	2.53082673	1.33960874	0.003991

**Table 2:** PTEN target prediction scores of identified miRs. DIANA TOOLS miTG prediction score of the probability that the identified miRs will target PTEN. The closer the miTG score is to 1.0, the higher the probability of targeting.

MicroRNA	Predicted Targeted 3′UTR Position of PTEN Transcript	miTG Score
hsa-miR-92a-3p	59–84; 2842–2865; 3987–4009	0.971
hsa-miR-181a-5p	1261–1287; 1869–1887; 2255–2282; 2289–2316; 2780–2801; 3923–3933; 4680–4700; 5114–5137; 5249–5275; 5881–5908; 6194–6220	0.882
hsa-miR-222-3p	180–205; 2668–2685; 4381–4408; 5908–5928	0.812
hsa-miR-221-3p	180–205; 2668–2685; 4381–4408; 5908–5928	0.755
hsa-miR-21-5p	1588–1607; 4789–4814	0.406

## Data Availability

The data presented in this study are available on request from the corresponding author.

## Supplementary Materials

The following supporting information can be downloaded at: https://www.mdpi.com/article/10.3390/cancers14112812/s1, Figure S1: Verification of Exosome Samples; Table S1: Cellular pathways and genetic targets of identified microRNAs; Figure S2: Western blot analysis of PTEN/p-AKT expression in cells where exosomes are retained or depleted from CAF-derived media.
