# Supplementary material for: Cancer-Associated Fibroblasts Confer Gemcitabine Resistance to Pancreatic Cancer Cells through PTEN-Targeting miRNAs in Exosomes
Source: Cancers (Basel). 2022 Jun 6;14(11):2812. doi: 10.3390/cancers14112812 (PMC9179363; doi:10.3390/cancers14112812)
Supplement: Supplementary file 1 [file cancers-14-02812-s001.zip › cancers-1652470-supplementary.pdf]

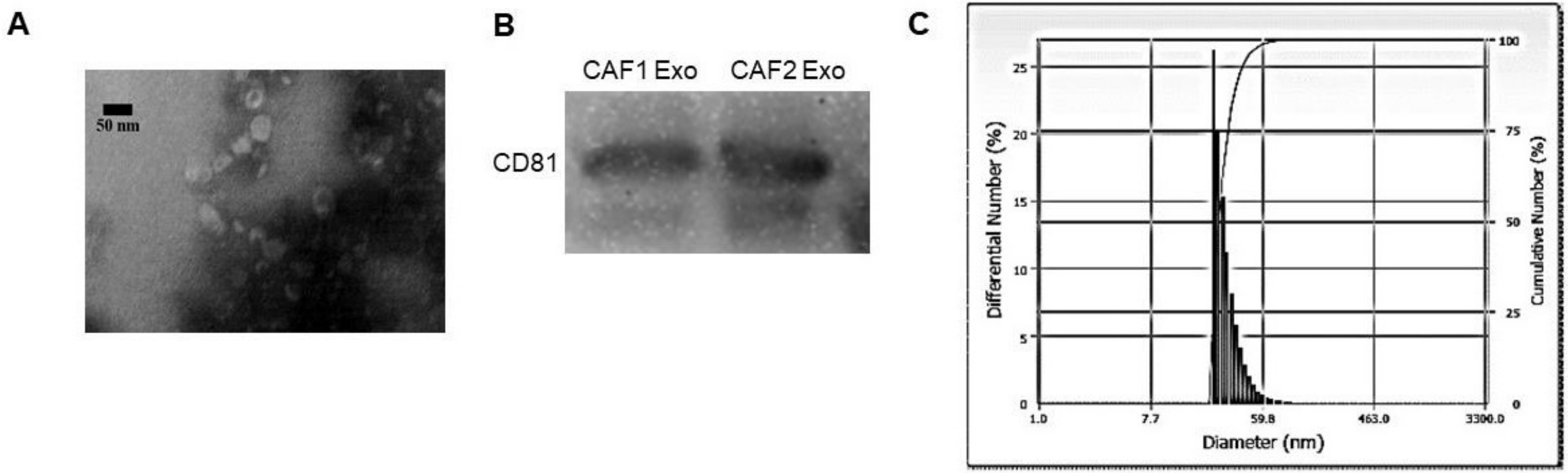

Supplemental Figure S1: **Verification of exosome samples.** CAF-secreted exosomes were analyzed for the correct shape and size via transmission electron microscopy (**a**) as well as for exosome protein markers such as CD81 (**b**). Average diameter of exosomes was analyzed via dynamic light scattering particle size analysis which showed a peak diameter size at 37nm (**c**).

Pathways Manipulated by all 5 microRNAs (miR-21- 5p, miR-92a-3p, miR-221- 3p, miR-181a-5p, and miR-222-3p)	Putative Gene Targets
**Wnt Signaling Pathway**	GSK3B, PPP2R5E, LRP6, TCF4, WNT5A, FZD6, SKP1, FRAT2, CAMK2A, NLK, SENP2, FZD10, AXIN2, CXXC4, WIF1, NFATC3, DAAM1, TBL1XR1
**MAPK Signaling Pathway**	FOS, NTF3, RASA2, CRK, CACNB4, RAP1A, FASLG, TAOK1, CACNA1I, NLK, DUSP10, RASGRP1, PPM1A, DUSP8, CDC42, FGF18, RPS6KA3, AKT3, MAP2K1, STMN1, MAP3K2, MEF2C, DUSP5, MAP2K4, RAP1B, TGFBR2
**PI3K-AKT Signaling**	PHLPP2, PRLR, GSK3B, TSC1, PPP2R5E, ITGA8, PIK3CB, CREB5, YWHAG, COL27A1, ANGPT2, ITGA5, PIK3AP1, CDKN1B, FASLG, ITGAV, DDIT4, PIK3R3, COL5A1, KIT, PIK3R1, JAK3, FGF18, COL1A2, AKT3, PIK3CA, MAP2K1, ITGA6, PTEN, SGK3, KDR, SPP1, BCL2L11, RPS6KB1, COL4A1, IL6R
**HIF-1 Signaling**	STAT3, PIK3CB, ARNT, ANGPT2, CDKN1B, CAMK2A, PIK3R3, PIK3R1, AKT3, PIK3CA, MAP2K1, PDHB, PFKFB4, RPS6KB1, EGLN1, IL6R
**Focal Adhesion**	GSK3B, CRK, ITGA8, PIK3CB, PIP5K1C, RAP1A, COL27A1, ITGA5, VCL, ITGAV, PPP1R12A, PIK3R3, COL5A1, PIK3R1 CDC42, COL1A2, AKT3, PIK3CA, MAP2K1, ITGA6, PTEN, KDR, RAP1B, SPP1, COL4A1
**T cell Receptor Signaling**	FOS, GSK3B, PIK3CB, PIK3R3, RASGRP1, PIK3R1, CDC42, AKT3, CD4, PIK3CA, MALT1, MAP2K1, CARD11, NFATC3
**VEGF Signaling**	PIK3CB, PIK3R3, PTGS2, PIK3R1, CDC42, AKT3, PIK3CA, MAP2K1, NFATC3, KDR
**Endocytosis**	CHMP7, DNAJC6, GRK5, GRK7, PIP5K1C, PDCD6IP, EEA1, ITCH, VPS36, ASAP1, ZFYVE16, RAB11FIP2, GIT2, SMURF1, RAB11A, KIT, CDC42, SMAD7, RAB11FIP1, KDR, TGFBR2, ADRB1
**Jak-Stat Pathway**	PRLR, STAT3, PIK3CB, CSF2RB, CNTFR, SPRED2, PIK3R3, LIFR, PIK3R1, JAK3, SPRY1, PIAS4, AKT3, PIK3CA, SPRY2, IL6R
**Endoplasmic Reticulum Protein Processing**	UBE2E3, SAR1B, YOD1, HSPA5, UBE2J1, SKP1, EDEM1, EDEM3, MAN1A2, SVIP, SEC62, SEC24A, LMAN1, UBE2D3, SEC24B, UBE2G1, DNAJB12, DERL1, ATXN3, RRBP1, PARK2
**Ubiquitin Mediated Proteolysis**	UBE2E3, WWP2, FBXW7, ITCH, UBE2J1, SKP1, CUL5, SKP2, BIRC6, SMURF1, PIAS4, UBE2D3, CDC27, UBE2G1, UBE2W, PARK2
**B Cell Receptor Signaling**	FOS, GSK3B, PIK3CB, PIK3AP1, PIK3R3, PIK3R1, AKT3, PIK3CA, MALT1, MAP2K1, CARD11, NFATC3

Supplemental Table S1

Cellular pathways and genetic targets of identified microRNAs.

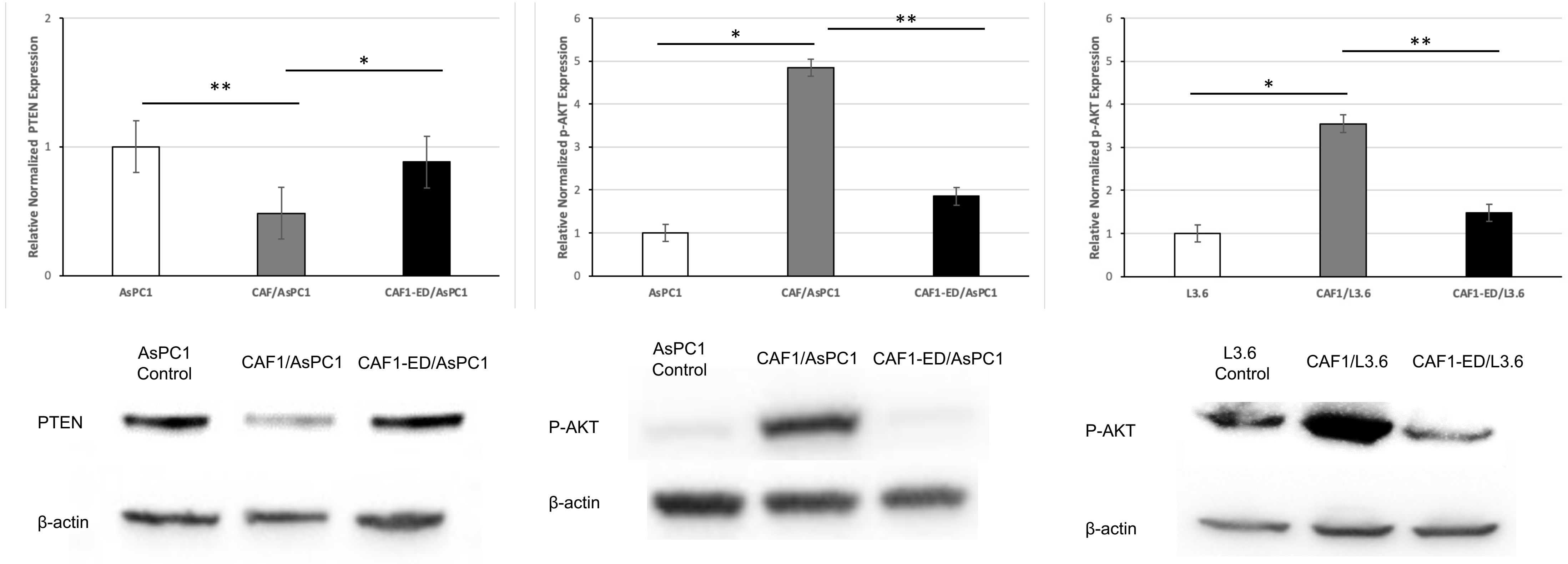

Supplemental Figure S2: Western blot analysis of PTEN/p-AKT expression in cells where exosomes are retained or depleted from CAF-derived media. (Top row) Quantification of relative PTEN protein levels or phosphorylated AKT protein levels (p-AKT) within cell lysates of epithelial cells cultured in normal media (control), CAF-conditioned media, or exosome-depleted CAF-conditioned media (CAF1-ED). (Bottom Row) Representative images of western blots used for protein quantification. Protein levels were normalized and quantified using ImageJ software. ***p*<0.01. **p*<0.05.
